# Beyond the tumor: the role of the gut microbiome in triple-negative breast cancer

**DOI:** 10.3389/fonc.2026.1832176

**Published:** 2026-05-22

**Authors:** Armina Saadatkhah, Laura Nicholson, Thomas A. Buchholz, Lee Hong

**Affiliations:** 1 University of California, San Diego, San Diego, CA, United States; 2 Department of Translational Medicine, Scripps Research, La Jolla, CA, United States; 3 Scripps Cancer Center, Scripps Health, San Diego, CA, United States; 4 Clinical Assistant Professor, Department of Medical Oncology and Therapeutics Research, City of Hope Lennar Foundation Cancer Center, Irvine, CA, United States

**Keywords:** chemo-immunotherapy, dysbiosis, gut microbiome, short-chain fatty acids, triple negative breast cancer, tumor microenvironment

## Abstract

The gut microbiome, a metabolically active community of microorganisms in the gastrointestinal tract, regulates host immunity, metabolism, and inflammation. Dysbiosis, or disruption of this ecosystem, has been linked to cancer initiation, progression, and therapy resistance. Triple-negative breast cancer (TNBC) accounts for 10–15% of breast cancers and is managed primarily with chemotherapy and immune checkpoint inhibitors; however, treatment responses remain variable and these patients are at higher risk of cancer recurrence compared to patients with hormone receptor-positive or HER2-positive breast cancer. Emerging evidence suggests that the gut microbial composition and its diversity can influence outcomes and therapeutic efficacy of systemic treatments in TNBC. We review the current epidemiologic, mechanistic, and clinical evidence on how the gut microbiome influences TNBC biology, with particular attention to the tumor immune microenvironment and response to therapy. We highlight protective and pro-tumorigenic microbial signatures, the impact of antibiotics and obesity, and emerging strategies, such as dietary modulation and microbiome-targeted interventions, that may ultimately be used to optimize TNBC management and improve patient outcomes.

## 1 Introduction

Breast cancer is the most common malignancy in women worldwide. Among its molecular subtypes, triple-negative breast cancer (TNBC) is one of the most aggressive, accounting for 10–15% of cases. Clinically, TNBC is characterized by high histologic grade, rapid progression, early metastasis, and poorer survival compared with other breast cancer subtypes (1). TNBC lacks expression of the three key receptors – estrogen receptor (ER), progesterone receptor (PR), and hormone-epidermal growth factor receptor 2 (HER2) – rendering it unresponsive to endocrine therapies and HER2-targeted agents, leaving cytotoxic chemotherapy and immunotherapy as the main systemic treatment options (2, 3). Trials such as KEYNOTE-522 have demonstrated that the addition of immune checkpoint blockade to neoadjuvant chemotherapy significantly improves pathologic complete response (pCR) and event-free survival (EFS) in early-stage TNBC (4). These advances underscore the central role of the immune system in TNBC control and highlight the need to understand upstream regulators of antitumor immunity. Additionally, the KEYNOTE-355 trial demonstrates that adding pembrolizumab to chemotherapy significantly improves overall survival in patients with PD-L1–positive metastatic TNBC, defined as combined positive score (CPS) ≥ 10 (5).

Increasing attention has turned to the gut microbiome as a critical regulator of host immunity, systemic inflammation, and treatment response in multiple cancers, including breast cancer (6–10). Trillions of microorganisms inhabit the gastrointestinal tract, where they collectively regulate mucosal and systemic immune responses, epithelial barrier integrity, and metabolic homeostasis (8). This ecosystem spans a continuum between eubiosis, defined by high microbial diversity along with functional balance, and dysbiosis, an imbalanced and pro-inflammatory state (8–11).

In TNBC, several lines of evidence suggest that gut microbial composition and diversity influence tumor biology and the efficacy of systemic therapies, particularly chemo-immunotherapy. Higher microbial diversity and enrichment of short chain fatty acids (SCFA)- producing taxa have been associated with improved treatment responses, whereas dysbiosis, often driven by obesity, antibiotic exposure, and environmental factors, is linked to chronic inflammation, immunosuppression, and worse outcomes (9, 12–17).

Beyond the gut, there is growing recognition that tumors themselves harbor distinct intratumoral microbiomes, microbial communities that colonize breast tissue through hematogenous spread, gut translocation through a disrupted intestinal barrier, and local invasion from adjacent mucosal surfaces (18, 19) signaling.

In this review, we examine how gut microbiota influence TNBC risk, immune regulation, and response to neoadjuvant chemo-immunotherapy, integrating concepts of eubiosis and dysbiosis with epidemiologic factors, and emerging microbiome-based therapeutic strategies.

## 2 The gut microbiome, eubiosis, dysbiosis, and the intestinal barrier

**Figure 1:**
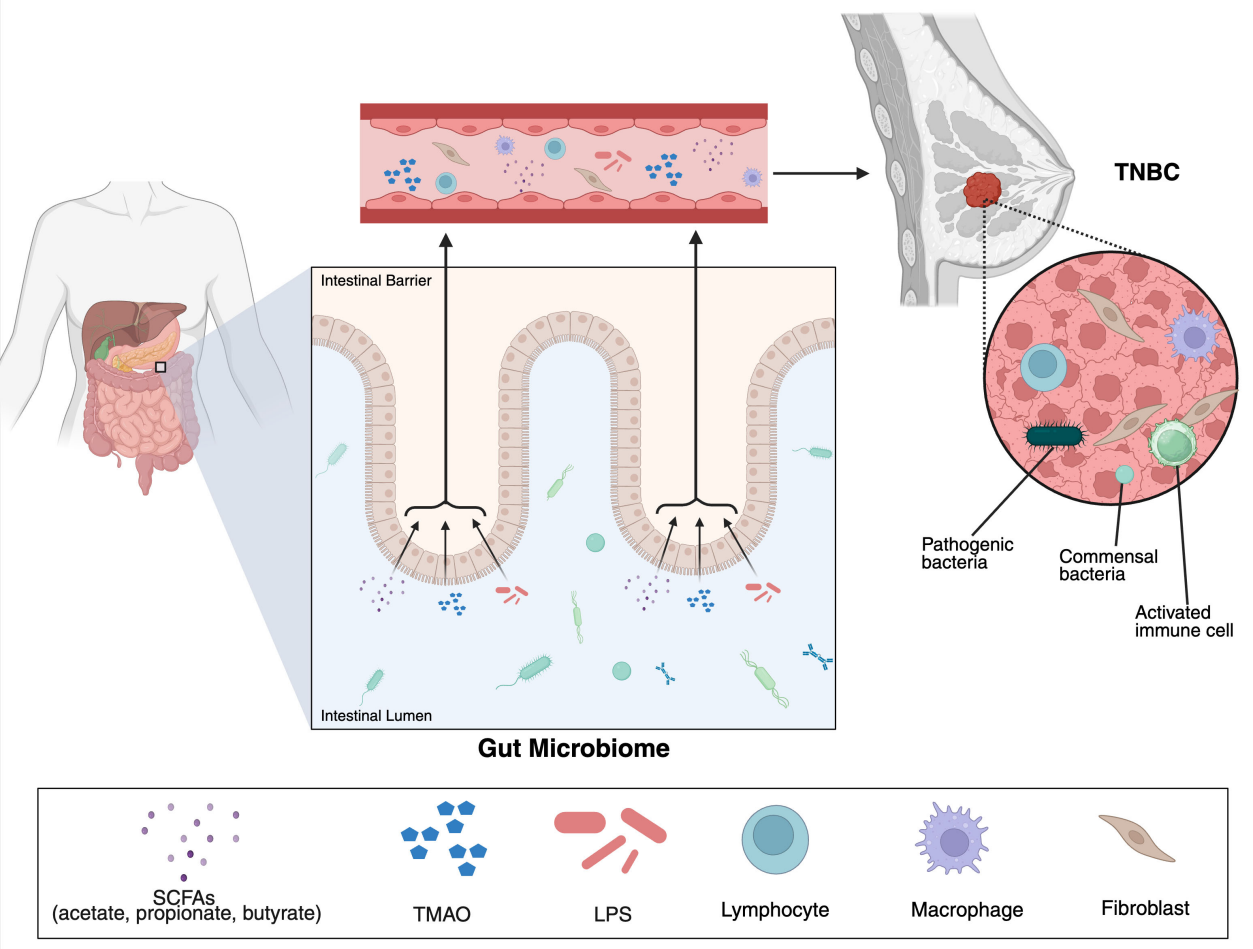
Gut microbiome-derived metabolites influence the TNBC tumor microenvironment. Gut bacteria in the intestinal lumen produce key metabolites, including short-chain fatty acids (acetate, propionate, and butyrate), trimethylamine N-oxide (TMAO), and lipopolysaccharide (LPS). These metabolites cross the intestinal barrier, enter systemic circulation, and travel to distant sites including the breast tumor. Within the TNBC tumor microenvironment, translocated pathogenic and commensal bacteria interact with immune cells (lymphocytes and macrophages) and stromal fibroblasts to shape the local inflammatory landscape. This gut–tumor axis suggests that imbalances in the gut microbiome may remotely influence immune infiltration, stromal remodeling, and tumor progression in TNBC.

The gut microbiome forms a dense, dynamic community that performs essential metabolic, immunologic, and homeostatic functions (8). In a state of eubiosis, microbial diversity is high, and beneficial taxa such as *Akkermansia*, *Faecalibacterium*, and *Bifidobacterium*, along with members of the *Ruminococcaceae* and *Lachnospiraceae* families, are abundant. These organisms produce SCFAs, including butyrate, propionate, and acetate, which reinforce epithelial barrier integrity, regulate mucosal immunity, suppress pathogen overgrowth, and limit chronic inflammation (8, 11, 13, 20, 21). Under eubiotic conditions, the intestinal barrier, composed of 1) luminal microorganisms and their metabolites, 2) a protective mucus layer, 3) epithelial cells joined by tight junctions, 4) a subepithelial immune network, and 5) the vasculature, maintains selective permeability along with balanced immune tolerance (8, 11, 21). In contrast, dysbiosis is characterized by reduced microbial diversity and an overrepresentation of potentially pathogenic or pro-inflammatory taxa (9, 15, 22). This dysbiotic shift disrupts the intestinal barrier through mechanisms including weakened tight junctions, thinning of the mucus layer, and increasing intestinal permeability. As a result, microbial products such as lipopolysaccharide (LPS) translocate into the circulation, driving systemic inflammation and immune dysfunction (11, 14). Once in the bloodstream, microbial metabolites and inflammatory mediators can reach distant organs, including breast tissue, where they can alter local immune responses and create a pro-tumorigenic environment (**Figure 1**). Collectively, these mechanisms provide a clear link for how gut dysbiosis contributes to carcinogenesis and therapy resistance in TNBC (8, 14, 21).

## 3 Epidemiologic risk factors intersecting with the microbiome

Obesity is among the strongest modifiable risk factors for TNBC, since overweight and obese women tend to have higher risk of TNBC, with particularly strong associations in postmenopausal women, along with clear effects in younger populations as well. Obesity promotes chronic low-grade inflammation, insulin resistance, and altered adipokine signaling; it also reshapes gut microbiota by favoring pro-inflammatory taxa and depleting SCFA producers (23–26). These alterations increase circulating LPS and bile acids (23), and activate NF-κB (23–25), and STAT3 signaling pathways (25), all of which contribute to an immunosuppressive tumor microenvironment (TME) (14).

Experimental data suggest that obesity-modulated microbiota can alter TNBC growth, immune infiltration, and response to therapy. In TNBC mouse models (26), obesity reduces gut microbiome alpha diversity in both tumor-free and tumor-bearing states, while also altering taxonomic composition by decreasing *Bacteroides* (notably *Alistipes*) and enriching Firmicutes such as *Clostridia* and the family *Mogibacteriaceae*. Obese mice also show a relative increase in *Verrucomicrobia*, particularly *Akkermansia muciniphila*, which predominates in obesity but declines with tumor presence. Consistent with these findings, a systematic review of 391 breast cancer studies (23) found that obesity is associated with poorer overall and breast cancer-specific survival in both pre- and postmenopausal women with hormone receptor–positive disease, although the prognostic impact of obesity varies across studies.

Another key epidemiologic context that may affect the role of the gut microbiome is racial disparity. TNBC disproportionately affects women of African descent, who experience higher incidence and worse outcomes than non-Hispanic white women (27–29). African ancestry–associated gene expression profiles in TNBC demonstrate distinct immune and metabolic signatures that may underlie more aggressive tumor biology and differential treatment responses (29, 30). Genetic variants related to West African ancestry have been linked to increased TNBC risk and poorer prognosis (31). Non-biologic factors, including socioeconomic disadvantage, chronic stress, structural racism, comorbid metabolic disease, and barriers to timely care also contribute to these disparities (29–31). Population-level differences in diet, environmental exposures, and microbiome composition likely intersect with these biologic and social determinants; however, direct evidence linking gut microbiota to racial differences in TNBC outcomes remains limited and largely speculative at present, representing a major gap in current research (14, 32, 33).

## 4 Protective mechanisms of gut microbiome in cancer

Several bacterial taxa appear to confer protective, antitumor effects. The gut microbiome shapes systemic antitumor immunity by regulating dendritic cell maturation, T-cell priming, and cytokine profiles. *Akkermansia muciniphila* enhances dendritic cell activation, maintains gut barrier integrity, and correlates with improved immunotherapy responsiveness (25). Recent mouse colonization experiments demonstrate that autoinducer-2 (AI−2) produced by *Blautia obeum* promotes *Akkermansia muciniphila* colonization, suggesting a cell-density dependent protective marker (34). Additional species, including *Bifidobacterium longum*, *Bacteroides fragilis*, and members of the *Ruminococcaceae* family, have been associated with improved efficacy of immune checkpoint inhibitors across multiple cancers.

Microbial metabolites, especially SCFAs, function both as histone deacetylase inhibitors and as signaling molecules that regulate T-cell differentiation, along with antibody effector function (24, 35). SCFA-producing organisms such as *Faecalibacterium prausnitzii*, *Eubacterium rectale*, and *Ruminococcus* generate butyrate and related metabolites that suppress inflammation, enhance CD8+ T cell activity, and promote M1 macrophage polarization, collectively supporting antitumor immunity (36). As illustrated in **Figure 1**, gut- derived metabolites, such as SCFAs, trimethylamine N-oxide (TMAO), and LPS, cross the intestinal barrier, enter systemic circulation, and travel to the breast tumor, where translocated bacteria, immune cells, and fibroblasts collectively shape the TNBC microenvironment. Together, these findings highlight how restoring beneficial microbial balance may enhance therapeutic outcomes and partially mitigate existing TNBC differences (12–14, 20, 22, 37). Across several TNBC cohorts treated with neoadjuvant chemotherapy, higher baseline gut microbial diversity has consistently been associated with higher pCR rates (12, 37–39). Responders typically demonstrate enrichment of SCFA-producing taxa such as *Faecalibacterium*, *Ruminococcaceae*, *Lachnospiraceae*, *Bacteroides* and *Eubacterium*, along with metabolite signatures in amino acid and tryptophan pathways that correlate with elevated tumor-infiltrating lymphocytes and an inflamed TME (32, 37–39). Enrichment of SCFA-producing taxa also correlates with longer progression-free survival and enhanced systemic immune activation (16, 37).

Within tumors, greater microbial richness is associated with increased infiltration of CD4^+^ T cells, elevated CXCL13 expression (38, 39), and more mature tertiary lymphoid structures (39) that act as local immune hubs supporting antitumor responses. These tumors also tend to have fewer M2-like macrophages, which typically suppress immunity and promote tumor progression (38). Microbial metabolites further contribute to TME modulation. For example, the microbial metabolite TMAO has been shown in TNBC models to enhance dendritic cell activation, increase CD8^+^ T-cell infiltration, slow tumor growth, and improve response to immune checkpoint blockade, whereas depletion of TMAO-producing bacteria abrogates these benefits (40–42).

## 5 Pro tumorigenic mechanisms

Conversely, several microbial taxa exhibit pro-tumorigenic characteristics. Pathogens including *Escherichia coli*, *Fusobacterium nucleatum*, *Staphylococcus aureus*, and *Staphylococcus epidermidis* can produce genotoxins, impair epithelial barrier function, and generate inflammatory microenvironments that facilitate tumor growth and metastasis (9, 10). Gut and tumor samples from TNBC patients are often enriched in *Enterobacteriaceae*, *Actinomycetaceae*, and *Sphingobacteriaceae*, and reduced in *Sphingomonas*, a beneficial taxon that normally activates natural killer T cells (6). Compared with healthy controls, breast cancer patients tend to display greater abundance of *Enterobacteriaceae*, *Staphylococcus*, and *Bacillus*, further reinforcing the link between microbial imbalance, chronic inflammation, and cancer progression (43). **Table 1** outlines each taxon’s role in TNBC biology, its primary functions within the gut microbiome (including short-chain fatty acid production, barrier maintenance, and immune modulation), and notes whether it is generally associated with protective or pro-tumorigenic effects.

**Table 1:** Key gut bacterial taxa implicated in triple-negative breast cancer (TNBC).

Bacteria	Beneficial vs harmful	Role in TNBC	Function in gut microbiome
*Akkermansia muciniphila*	Beneficial	Enhances immune activation and contributes to improving chemo-immunotherapy response	Maintains the protective mucin layer barrier and produces beneficial SCFAs
*Faecalibacterium prausnitzii*	Beneficial	Produces SCFAs serving as a marker of eubiosis; supports CD8+ T cells, contributing to antitumor immunity	Produces anti-inflammatory butyrate
*Ruminococcus*	Beneficial	Supports systemic immune modulation and antitumor immunity by SCFAs	Ferments fiber and produces butyrate
*Bifidobacterium longum*	Beneficial	Enhances immunotherapy outcomes; often included in probiotic studies	Produces SCFA and supports immunity
*Bacteroides fragilis*	Beneficial	Enhances immunotherapy response	Metabolizes polysaccharides and modulates immunity
*Escherichia coli*	Harmful	Produces genotoxins, such as colibactin, that promote tumor progression	Overgrows opportunistically, disrupting microbiome homeostasis
*Fusobacterium nucleatum*	Harmful	Associates with pro-tumor inflammation and tumor progression	Disrupts epithelial barrier through pro-inflammatory activity

Disruption of gut microbial balance has profound immunologic consequences.

Antibiotic exposure depletes commensal bacteria that support immune homeostasis (44), leading to reduced SCFA production (44), impaired dendritic cell priming (15), diminished lymphocyte levels, and weakened systemic immunity (15, 44).

Dysbiosis also increases intestinal permeability, facilitating translocation of microbial products and enhanced TLR4 signaling on tumor-associated macrophages (14, 45). This signaling promotes polarization toward an M2-like immunosuppressive phenotype, reinforcing immune suppression within the TME (39, 46). These immunobiological effects translate into clinically meaningful differences in treatment response and survival.

Ransohoff et al. (15) studied 772 women with stage I-III TNBC diagnosed between 2000–2014 and treated with curative intent prior to the immune checkpoint inhibitor era. Only antimicrobials prescribed after breast cancer diagnosis were analyzed. No single antimicrobial class drove the association with worse outcomes, rather it was the cumulative quantity and diversity of unique drug classes that mattered. Each additional month of antibiotic was associated with a 5% increase in the hazard ratios of breast cancer-specific survival and overall survival (HR 1.05 for both). The effect was sustained through year three post-diagnosis before attenuating, and statistical modeling suggests this impact was mediated through reductions in peripheral lymphocyte count rather than neutrophil count. In support of this correlation, non-responders tend to exhibit lower baseline diversity, depletion of beneficial taxa during therapy, and enrichment of potentially pathogenic bacteria, consistent with impaired immune regulation and a more immunosuppressive microenvironment (32, 33, 47).

Evidence from the metastatic setting further supports these associations. In the ALICE trial, patients with metastatic TNBC treated with immunomodulating chemotherapy plus the PD-L1 inhibitor atezolizumab (37) showed improved progression-free survival (PFS) compared with chemotherapy alone regardless of PD-L1 tumor status (16). Analyses of baseline gut microbiota demonstrated that high gut microbial alpha diversity, measured by Faith’s phylogenetic diversity, was associated with prolonged PFS (16), and predicted benefit from the atezolizumab-chemotherapy combination, whereas low diversity did not. These results underscore that patients with higher PD−L1 expression plus high gut microbial diversity derive the greatest benefit from checkpoint inhibition, highlighting how immune−checkpoint blockers interact with microbiomes to reshape the tumor microenvironment and improve outcomes in metastatic TNBC.

## 6 Role of intratumoral microbiome

Recent research into the breast cancer microenvironment has shifted the paradigm from viewing tumors as sterile sites to recognizing them as hosts to a dynamic, diverse, intratumoral microbiota. While the gut microbiome exerts significant influence through systemic metabolite and immune signaling through the “gut-mammary axis”, intratumoral bacteria establish residence through distinct physiological mechanisms (48). These microbes, often originating from commensal populations in the gut or oral cavity, colonize breast tissue through several routes, including translocation from the gut through a compromised intestinal mucosal barrier, hematogenous during transient, low-level bacteremia, and local invasion from adjacent skin or nipple-ductal surfaces (19, 48). This colonization is not passive; specific commensal organisms, such as *Fusobacterium nucleatum* and *Staphylococcus epidermidis*, are preferentially enriched in tumor tissues, where they modulate local DNA damage and dampen anti-tumor immune responses (48, 49). These intratumoral communities exist in direct physical proximity to cancer cells and tumor-infiltrating immune populations, positioning them as proximal regulators of the local tumor microenvironment (TME) (18, 48). Collectively, these findings suggest that the intratumoral microbiota functions as an active participant in tumor biology (19, 48).

## 7 Emerging microbiome-based strategies and future directions

This narrative review highlights how the gut microbiome influences triple-negative breast cancer (TNBC) biology and treatment response. Higher microbial diversity and short-chain fatty acid producing bacteria support anti-tumor immunity and better therapy outcomes, whereas dysbiosis from factors like obesity, diet, and antibiotics promotes an immunosuppressive tumor microenvironment that reduces treatment effectiveness.

Building on this framework, three complementary approaches may leverage microbiome insights in TNBC:

The first centers on dietary modification, one of the most accessible interventions. High-fiber diets have shown to expand SCFA-producing taxa, enhance gut barrier integrity, reduce systemic inflammation, and potentially improve antitumor immune responses (30, 44, 50). While TNBC-specific dietary clinical trials remain limited, this approach is mechanistically grounded and supported by epidemiologic and translational evidence. Ongoing work at our center is exploring structured high-fiber dietary interventions in early-stage TNBC, measuring microbiome composition alongside mood, activity, recurrence, and survival.

A second strategy that focuses on more direct microbiome interventions includes probiotics and fecal microbiota transplantation (FMT). These approaches aim to restore beneficial microbial functions and improve responsiveness to cancer therapies, particularly immunotherapy. Reviews of the TNBC microbiome literature identify these approaches as promising but note that supporting evidence is largely preclinical or extrapolated from other cancer types (51). A review in the International Journal of Cancer (52) highlights active investigation of FMT in immunotherapy, while a systematic review in Cancer Treatment Reviews (53) reports that FMT appears safe and feasible during cancer treatment though lacks conclusive TNBC-specific randomized trial data. Consequently, probiotics, prebiotics, and FMT remain experimental in TNBC and require validation through well-designed clinical studies before integration into standard practice.

Third, emerging strategies targeting the intratumoral microbiome directly offer additional avenues that may work synergistically with gut-focused approaches. These include nanoplatform-based antibacterial agents with dual antibacterial and anticancer activity, bacterial vaccines designed to activate dendritic cells against tumor-resident pathogens, and while bacteriophage therapy which enables highly specific targeting of pathogenic intratumoral organisms (18). Genetically engineered bacteria are also under investigation as delivery vehicles for immunostimulatory payloads directly within the tumor. These approaches collectively aim to reduce chemoresistance, alleviate treatment toxicity, and enhance immunotherapy efficacy, specifically by boosting CD8^+^ T-cell activation and potentiating PD-L1 blockade (18, 19, 49).

A fourth application involves integrating microbial profiles with imaging, immunologic, and metabolic data to develop predictive biomarkers. Evidence from other cancers (54) suggests that baseline microbiota composition can predict both immunotherapy efficacy and treatment-related toxicity. Microbiome research also informs risk mitigation during therapy, as unnecessary antimicrobial exposure disrupts microbial diversity and alters treatment outcomes. Avoiding unnecessary antibiotic use therefore represents one of the most immediately actionable strategies to preserve microbial diversity and potentially enhance treatment responsiveness, highlighting the importance of antibiotic stewardship in optimized cancer care (14, 54–57).

## 8 Limitations and safety considerations

Despite strong mechanistic rationale, deliberate microbiome modulation has not yet been established to produce meaningful improvement of TNBC outcomes, and several methodological limitations must be acknowledged. First, current studies are largely associative, heterogeneous, and limited by small sample sizes and cross-sectional designs. Significant variation exists across studies in patient populations (including differences in stage, age, genomic ancestry, comorbidities, and treatment setting). Variation in neoadjuvant chemotherapy and immunotherapy regimens across trials further compounds this heterogeneity, as different cytotoxic agents and immunotherapy combinations exert distinct effects on the gut microbiome. Differences in microbiome sampling methods (fecal versus mucosal versus intratumoral sampling) and sequencing platforms introduce technical variability that makes direct comparisons across cohorts challenging, as these methods differ substantially in taxonomic resolution and sensitivity. Additionally, studies have confounding variables, such as diet, body mass index, prior antibiotic use, metabolic comorbidities, and socioeconomic factors that independently influence both microbiome composition and cancer outcomes, which are not uniformly captured. Future progress will require rigorously designed, TNBC-specific prospective clinical trials with standardized microbiome collection protocols and integration of microbiome science with immunology, metabolism, and health-equity research to identify modifiable pathways capable of improving outcomes in this aggressive disease.

Microbiome targeted interventions, such as FMT and probiotics, carry clinically important risks that must be carefully considered, particularly in TNBC patients who are often immunocompromised during chemotherapy. FMT carries risk of bacteremia which can lead to sepsis and death. Additionally, FMT may facilitate the transfer of antibiotic resistance genes from donor to recipient microbiome, raising concerns about long-term antimicrobial resistance. Donor screening, standardized preparation protocols, and rigorous patient selection criteria are therefore essential. Probiotic use in immunocompromised patients also poses a risk of bacteremia or fungemia, particularly with Lactobacillus species in patients with central venous catheters or mucosal barrier disruption. These safety considerations underscore the need for dedicated clinical trials with appropriate adverse event monitoring before these strategies can be routinely recommended in the TNBC setting.

## 9 Conclusion

The gut microbiome is emerging as a critical and potentially modifiable determinant of TNBC risk and response to systemic therapy. TNBC patients frequently exhibit distinct gut and tumor microbial signatures characterized by reduced alpha diversity and enrichment of pro-inflammatory taxa, among. Higher microbial diversity and enrichment of SCFA-producing taxa correlate with improved chemo-immunotherapy responses, more inflamed TMEs, and better survival.

Microbiome-targeted strategies, including high-fiber diets, probiotics, fecal microbiota transplantation, and integrative biomarker platforms, offer promising avenues to enhance treatment efficacy. Ongoing and future trials, including structured dietary interventions and microbiome–immunotherapy combinations, will be essential to determine whether purposeful modulation of the microbiome can translate into improved outcomes for patients with TNBC. Until such data are available, careful antibiotic stewardship represent the most practical implications for TNBC care. Integrating microbiome science into TNBC research and clinical pathways has the potential to expand therapeutic efficacy and improve clinical outcomes in a breast cancer subtype that urgently requires more effective and equitable treatments.
